# Acute Respiratory Distress Syndrome (ARDS): Pathophysiological Insights and Lung Imaging

**DOI:** 10.3390/jcm8122171

**Published:** 2019-12-09

**Authors:** Gaetano Perchiazzi, Hermann Wrigge

**Affiliations:** 1 Hedenstierna Laboratory, Department of Surgical Sciences, Uppsala University, 75185 Uppsala, Sweden; 2 Department of Anesthesia and Intensive Care Medicine, Uppsala University Hospital, 75185 Uppsala, Sweden; 3 Department of Anesthesiology and Intensive Care Medicine, University Hospital Leipzig, 04103 Leipzig, Germany; hermann.wrigge@bergmannstrost.de; 4 Department of Anesthesiology, Intensive Care and Emergency Medicine, Pain Therapy, Bergmannstrost Hospital Halle, 06112 Halle, Germany

**Keywords:** mechanical ventilation, acute respiratory distress syndrome, imaging

## Abstract

Acute respiratory distress syndrome (ARDS) is in the center of the scientific debate both for its complex pathophysiology and for the discussion about the remedies that could contribute to its healing. The intricate interplay of different body systems that characterizes ARDS is mirrored by two main research threads, one centered on the pathophysiological mechanisms of the disease and the other on the new approaches to lung imaging. In this Special Issue of the *Journal of Clinical Medicine* are presented studies using imaging technologies based on electrical impedance tomography, synchrotron radiation computed tomography and intravital probe-based confocal laser endomicroscopy. The studies on the pathophysiological mechanisms pertain to the evaluation of the biomarkers of the disease and the platelet disfunction during extracorporeal membrane oxygenation. These contributions witness the intensity of ARDS research as many of the key problems of the disease are only in part resolved.

The threads that go through this Special Issue of the *Journal of Clinical Medicine* are devoted to clarifying some of the recent advances in pathophysiology and lung imaging of acute respiratory distress syndrome (ARDS). This syndrome, although recognized 50 years ago [1], is still in the center of the scientific debate both for its complex pathophysiology [2] and for the discussion about the remedies that could contribute to its healing [3]. It has been recently redefined [4] in the attempt of facilitating its prompt recognition and aligning the possible interventions to its clinical profile [5]. The complex interplay of different body systems renders ARDS, in the words of famous intensivists, “a textbook of intensive care medicine” [6]. This complexity is well mirrored by the contributions to the present number of the *Journal of Clinical Medicine*. Two different threads are followed, one centered on the pathophysiological mechanisms and implications of the disease and the other to new approaches to lung imaging during ARDS.

How important imaging is for recognition and management of ARDS is easily understandable by the fact that since the beginning of its identification, a method of lung imaging has been always used to define its presence [7]. In this issue of the *Journal of Clinical Medicine*, experts of the field proposed contributions to new approaches to lung imaging.

Putensen et al. [8] reviewed the technique of electrical impedance tomography (EIT) during cardiopulmonary monitoring. This interesting review spans from the physical basis of the method to image reconstruction and analysis. An important section is devoted to functional monitoring of both ventilation and perfusion. Of clinical interest is the review of the available literature behind all the functional measurements that render this paper a reference for EIT users. 

Haase et al. [9] have used quantitative computed tomography (CT) and electrical impedance tomography to assess recruitment and derecruitment in a porcine model of acid-aspiration-induced ARDS. Although this last can be considered a poorly recruitable model, methods based on individually-titrated Positive end-Expiratory Pressure (PEEP) can reduce tidal recruitment–derecruitment: noteworthily, these PEEP levels exceed the ones deriving from the ARDSnet table [10]. An important message for clinical practice is conveyed by the paper of Haase et al.: the use of EIT-derived information may permit to identify the patients who are able to respond to recruitment by reaeration of previously nonventilated lung regions. 

The clinical theme of tidal recruitment was also faced by Muders et al. [11] who aimed at using an EIT-derived measurement, the regional ventilation delay inhomogeneity index (RVDI), in the assessment of this phenomenon. RVDI was studied in an animal model of lung injury during slow inflation breaths, in order to define the applicability of this maneuver. The authors concluded that the combined use of EIT-RVDI with a slow inflation of 9 mL/kg body weight can adequately estimate tidal recruitment. 

A different imaging method has been studied by Scaramuzzo et al. [12]. Their paper is oriented to the definition of lung behavior during decremental PEEP application in a rabbit model of ARDS. In this case, the imaging method that is used is the synchrotron radiation computed tomography (SRCT) that allows the highest available resolution for tomographic methods in vivo. The study showed that in their model, the reduction in lung volume was related to a reduction of both the dimension and number of airspaces, although the dimensional reduction was the predominant mechanism. 

Remaining on the theme of the assessment of small lung structures, Lesur et al. [13] reviewed the technique named intravital probe-based confocal laser endomicroscopy that allows physicians to obtain accurate morphological information. The resolution provided by the method makes it possible to obtain a “virtual optical biopsy” of relevant clinical value in selected ARDS patients. 

The problem of diffuse alveolar damage (DAD) has been retrospectively investigated by Cardinal-Fernandez and colleagues [14]. The detection of this pathological finding requires a lung biopsy whose execution has inherent risks. For this reason, the authors wanted to understand whether DAD could be predicted by using clinical indicators as an alternative to biopsy. The result of the study showed that the presence of DAD could not be foreseen on the base of clinical variables, although DAD was significantly associated with in-hospital mortality. 

The biomarkers of acute lung injury were reviewed by Murray et al. [15], having as an ultimate goal the characterization of individualized approaches to ARDS. They focused on the most clinically relevant biomarkers of bronchoalveolar damage, endothelial damage, infection and inflammatory phenotype. In this respect, the recommendation by the authors was to integrate the information deriving from the observation of clinical phenotypes with the technologies of bioinformatics using a “reverse-translational” approach. 

The search for biomarkers of ARDS was also pursued by Kim et al. [16]. They focused on the exosomes from the brocholaveolar lavage (BAL) of patients affected by ARDS. They observed that although their level did not differ between subjects having different mortality at 28 days, it was negatively correlated with the ratio between PaO_2_/FiO_2_. 

Störmann and colleagues [17] studied a biomarker named club cell protein 16 (CC16) deriving from lung epithelial club cells that has anti-inflammatory tasks. The systemic concentrations of CC correlate with the extent of pulmonary contusion in traumatized patients. They evaluated whether, in a double-hit murine model (obtained by cecal ligation/puncture followed by chest trauma), the early neutralization of CC16 gave beneficial effects by abrogating the early immunosuppression of lung inflammation. They arrived to the conclusion that early local inhibition of CC16 reduced lung damage and had a protective effect. They reported also that a later inhibition of the same protein resulted in a worse pulmonary outcome. 

Wand and coauthors [18] explored the effects of extracorporeal (veno-venous) membrane oxygenation (ECMO) on platelet function assessed by multiple electrode aggregometry. They studied platelets at established time intervals from one hour before the beginning of ECMO till seven days after. They observed that platelet function in patients with severe ARDS is already impaired before the initiation ECMO. A further significant decrease in platelet aggregation estimation was found after 6 h, but all measurements recovered to baseline values on day two. The clinical advice by the authors, deriving from this study, is that platelet function should be assessed before initiation of ECMO and eventually corrected. 

Clinically relevant is the prospective study executed by Harnisch et al. [19] on the neurological outcome of 28 ARDS survivors. They discussed the fact that besides severe neurologic complications like intracranial hemorrhage and ischemic stroke, more subtle lesions detected by clinical examination are nevertheless sufficient to hamper daily activities, the quality of life and psychological health. They conclude that the burden of these complications needs to be weighed when indicating the ECMO treatment for patients affected by ARDS.

The contributions that the *Journal of Clinical Medicine* has hosted in this monographic number witness the vivacity and intensity of ARDS research. The complexity of ARDS warrants many years of further research as many of the key problems of the disease [20] are only in part resolved.
